# Relevance of the TAS’/PASP Ratio as a Predictor of Outcomes in Patients with Heart Failure with a Reduced Ejection Fraction

**DOI:** 10.3390/life14070863

**Published:** 2024-07-10

**Authors:** Ilija Srdanović, Maja Stefanović, Aleksandra Milovančev, Aleksandra Vulin, Teodora Pantić, Dragana Dabović, Snežana Tadić, Aleksandra Ilić, Anastazija Stojšić Milosavljević, Marija Bjelobrk, Tatjana Miljković, Lazar Velicki

**Affiliations:** 1 Faculty of Medicine, University in Novi Sad, 21000 Novi Sad, Serbia; ilija.srdanovic@mf.uns.ac.rs (I.S.); aleksandra.milovacev@mf.uns.ac.rs (A.M.); aleksandra.vulin@mf.uns.ac.rs (A.V.); dragana.dabovic@mf.uns.ac.rs (D.D.); snezana.tadic@mf.uns.ac.rs (S.T.); aleksandra.ilic@mf.uns.ac.rs (A.I.); anastazija.stojsic@mf.uns.ac.rs (A.S.M.); marija.bjelobrk@mf.uns.ac.rs (M.B.); tatjana.miljkovic@mf.uns.ac.rs (T.M.); lazar.velicki@mf.uns.ac.rs (L.V.); 2 Institute for Cardiovascular Diseases of Vojvodina, 21204 Sremska Kamenica, Serbia; teodora.pantic@ikvbv.ns.ac.rs

**Keywords:** coupling, prognosis, heart failure, right ventricle, pulmonary artery

## Abstract

Background: There is evidence that right ventricular (RV) contractile function, especially its coupling with the pulmonary circulation, has an important prognostic value in patients with left ventricular dysfunction. Aims: This study aimed to identify the best echocardiographic parameters of RV function and pulmonary artery systolic pressure (PASP) alone or in the form of the index of right ventricular-pulmonary artery coupling (RV-PA coupling) to determine the best predictor of 1-year major adverse cardiovascular events (MACE), which were defined as cardiovascular death and cardiac decompensation in heart failure patients with reduced ejection fraction (HFrEF). Methods and results: The study enrolled 191 HFrEF patients (mean age 62.28 ± 12.79 years, 74% males, mean left ventricular ejection fraction (LVEF) 25.53 ± 6.87%). All patients underwent clinical, laboratory, and transthoracic echocardiographic (TTE) evaluation, focusing on assessing RV function and non-invasive parameters of RV-PA coupling. RV function was evaluated using fractional area change (FAC), tricuspid annular plane systolic excursion (TAPSE), and peak tricuspid annular systolic velocity (TAS’). PASP was estimated by peak tricuspid regurgitation velocity (TRVmax) and corrected by assumed right atrial pressure relative to the dimension and collapsibility of the inferior vena cava. The TAPSE/PASP and TAS’/PASP ratios were taken as an index of RV-PA coupling. During the follow-up (mean period of 340 ± 84 days), 58.1% of patients met the composite endpoint. The independent predictors of one-year outcome were shown to be advanced age, atrial fibrillation, indexed left atrial systolic volume (LAVI), LVEF, TAPSE/PASP, and TAS’/PASP. TAS’/PASP emerged as the strongest independent predictor of prognosis, with a hazard ratio (HR) of 0.67 (0.531–0.840), *p* < 0.001. Reconstructing the ROC curve 0.8 (0.723–0.859), *p* < 0.001, we obtained a threshold value of TAS’/PASP ≤ 0.19 (cm/s/mm Hg) (sensitivity 74.0, specificity 75.2). Patients with TAS’/RVSP ≤ 0.19 have a worse prognosis (Log Rank *p* < 0.001). Conclusions: This study confirmed previously known independent predictors of adverse outcomes in patients with HfrEF—advanced age, atrial fibrillation, LAVI, and LVEF—but non-invasive parameters of RV-PA coupling TAPSE/PASP and TAS’/PASP improved risk stratification in patients with HFrEF. Variable TAS’/PASP has been shown to be the most powerful, independent predictor of one-year outcome.

## 1. Introduction

Ventricular-artery coupling indicates the efficiency of energy transfer from the ventricle to the arterial load. Research has shown the importance of left ventricular-arterial coupling in conditions such as hypertension and heart failure. Similarly, the coupling between the right ventricle and the pulmonary artery has been found to be crucial in various diseases, including pulmonary hypertension, heart failure, and valvular disease [1].

Right ventricular dysfunction often occurs as a result of various cardiopulmonary diseases and contributes to the progression of the underlying disease [2,3,4,5]. It is also an important determinant of adverse outcomes associated with the risk of developing cardiac death, and re-hospitalization due to repeated decompensation [6,7,8]. In normal conditions, the RV is coupled to low-pressure and high-compliance pulmonary circulation [9]. Pulmonary hypertension (PH) is a hemodynamic condition characterized by elevated mean pulmonary artery pressure (m-PAP) measured at rest with right heart catheterization (RHC). The ESC/ERS 2022 Guidelines have updated the hemodynamic definition of pulmonary hypertension (PH) by lowering the mean pulmonary artery pressure (mPAP) threshold from 25 to 20 mmHg. The threshold for peak tricuspid regurgitation velocity (TRVmax) remains unchanged (>2.8 m/s). However, the potential value of other echocardiographic variables in the diagnosis of PH according to the new criteria has not been thoroughly evaluated. PH due to left heart disease is the most common type of PH, accounting for 65–80% of cases [10,11]. Once PH develops, the pulmonary vascular bed becomes a high-pressure, high-resistance, and low-compliance system, leading to an additional load on the contracting ventricle and a disruption of the RV-PA coupling. Maladaptive RV hypertrophy and/or dilatation occur during the disease, leading to RV failure [12]. Assessment of RV function, especially RV-PA coupling, is an important prognostic marker in patients with heart failure (HF) and PH [13,14,15,16]. Right heart catheterization (RHC) is the gold standard for diagnosis of PH and assessment of RV-PA coupling but is expensive, technically demanding, and usually unavailable at the bedside or in critical care conditions and has risks that are not negligible. Non-invasive estimation of PASP using echocardiography has increased its sensitivity for quantifying PASP and highly correlates with invasive measurements in advanced HFrEF [10]. Right ventricular systolic function is evaluated by transthoracic echocardiography (TTE) using several parameters, including fractional area change (FAC), tricuspid annular plane systolic excursion (TAPSE), and tricuspid annular systolic velocity (TAS’) [17,18,19,20].

The TAPSE/PASP ratio is a non-invasive, indirect measurement of RV-PA coupling. It has been validated as an important clinical and prognostic parameter in patients with HF. Additionally, it is identified as an independent and strong predictor of outcome in patients with combined post- and pre-capillary PH, as well as in pulmonary arterial hypertension (PAH) [21,22,23,24,25]. Despite its potential clinical utility, the optimal cut-off value for TAPSE/PASP has yet to be determined and requires further investigation [13,14,15,21,22,23,25]. This study aimed to find the best echo parameters of RV function and RV-PA coupling, to predict a one-year composite endpoint of cardiovascular death and hospitalization due to cardiac decompensation in HFrEF patients. The study found that the TAPSE/PASP and TAS’/PASP variables are valuable indicators of RV-PA coupling and could predict adverse outcomes in HFrEF patients. Notably, the study also discovered that the TAS’/PASP ratio had not been previously evaluated as a predictor of adverse outcomes in HFrEF patients.

## 2. Materials and Methods

### 2.1. Study Population

This prospective study included 191 patients who were admitted to our university teaching hospital in the period from June 2017 to January 2019 due to symptomatic HFrEF and NYHA functional class III-IV. We conducted a one-year follow-up with patients to determine a composite endpoint, defined as cardiac death and hospitalization due to recurring cardiac decompensation. The composite endpoint was identified by accessing the hospital information system database and through telephone communication with patients or their family members. The study involved patients aged 18 and above who had been hospitalized due to HFrEF and NYHA class III-IV. Exclusion criteria comprised acute coronary events during or within six months before the index hospitalization, poor-quality echo imaging, and patients with a severe form of chronic obstructive pulmonary disease. The study classified heart failure etiology as non-ischemic or ischemic cardiomyopathy (CMP) based on the Felker definition of ischemic cardiomyopathy, which considers a prior medical history of MI, revascularization, or the presence of significant multi-vessel coronary disease, left main, or proximal LAD disease as indicative of ischemic CMP. Significant coronary artery disease was defined as confirmed stenosis of ≥50% in major coronary arteries validated by invasive or multi-slice computed (MSCT) coronary angiography [26].

### 2.2. Ethics

All participants were requested to provide written consent, which was duly obtained, and the investigation was conducted in accordance with the principles laid out in the Declaration of Helsinki. Additionally, the ethics committee of our institution granted its approval for the study, thereby ensuring that all ethical considerations were met.

### 2.3. Echocardiography

TTE was performed using the Vivid XD ultrasound system from GE Healthcare in Norway, in line with the European and American Society of Echocardiography’s recommendations. The echocardiography techniques that we used were 2-dimensional echocardiography, M-mode, Doppler echocardiography, and tissue Doppler imaging to evaluate the morphology and function of both the left and right ventricles [19,20]. The recordings were taken for at least three consecutive heartbeats in patients with normal sinus rhythm and for five to seven consecutive heartbeats in patients with atrial fibrillation (AF), and the average values were calculated. LVEF was calculated using the biplane Simpson method with apical 2- and 4-chamber views. Various modified views were used to obtain imaging and evaluate the condition of the right ventricle. To assess right heart morphology, we measured the RV end-diastolic dimension (RVEDD mm) and the right atrial area (RAA cm^2^). In addition, TAPSE (mm), TAS’ (cm/s), and the FAC (%) were measured to evaluate RV systolic function. TAPSE refers to the maximal systolic excursion of the lateral tricuspid annulus, which can be measured through M-Mode in an apical 4-chamber view. An RV dysfunction can be indicated by a TAPSE value of less than 17 mm. The TAS’ provides the longitudinal velocity of the tricuspid annular plane through tissue Doppler imaging. The sample volume was placed in the basal segment of the right ventricle’s free lateral wall. A TAS’ value of 9.5 cm/s indicates RV dysfunction. FAC measures the endocardial RV borders during end-diastole (Area ED) and end-systole (Area ES) and calculates the area change using this formula: 100 × (RV-Area ED − RV-Area ES)/RV-Area ED. A value of less than 35% indicates RV systolic dysfunction. To estimate the RV loading condition, we measured the TRVmax and assessed the PASP. The continuous wave Doppler (CWD) was used to determine the TRVmax (m/s), which was then used to calculate the pressure gradient between the right ventricle (RV) and right atrium (RA) using the modified Bernoulli equation (4 × TRVmax^2^). The PASP (mmHg) was obtained by adding the estimated right atrial pressure to the calculated pressure gradient between RV and RA, which was mentioned earlier: 4 × (TRVmax^2^) + estimated right atrial pressure. We estimated the right atrial pressure by considering the diameter and collapsibility of the inferior vena cava. To ensure accurate results, we only included patients who showed noticeable TR signals and had inspiratory/expiratory phases of inferior vena cava diameters. Measuring PASP over the maximum speed of tricuspid regurgitation provides only an estimate and not an exact value. Evaluating tricuspid regurgitation can be challenging due to poor ultrasound signals, but we can utilize echocardiography to measure PASP in most cases. However, in severe pulmonary hypertension, echocardiography may not always provide reliable results [20]. For the non-invasive evaluation of the RV-PA coupling, we evaluated two combined echocardiographic parameters, namely, TAPSE/PASP (mm/mmHg) and TAS’/PASP (cm/s/mmHg).

### 2.4. Statistic Methods

The present study assessed the normality of the distribution through the Kolmogorov–Smirnov test. The *t*-Test and Mann–Whitney were used to evaluate the differences in mean values of parameters, based on the normality of the distribution. The chi-square test (ꭓ^2^) and Fisher’s exact test were utilized to determine the disparities in the frequencies of individual parameters for groups with and without adverse outcomes. Univariate and multivariate Cox regression analyses were carried out to examine the relationship between the time to onset of the composite endpoint and different parameters. Kaplan–Meier curves were constructed to compare survival times for different levels of observed parameters, and differences were tested by the Log Rank test. ROC curves were used to determine the limit values of the observed parameters as a test to predict the occurrence of adverse outcomes. The area under the ROC curve was calculated to maximize the specificity and sensitivity of the test. The statistical analysis was performed using Microsoft Excel 2007 and the statistical package Statistics 13.3.1 (StatSoft Inc., Tulsa, OK, USA). The significance level was set at *p* < 0.05.

## 3. Results

The study enrolled 191 HFrEF patients (mean age 62.28 ± 12.79 years, 74% males, mean left ventricular ejection fraction (LVEF) 25.53 ± 6.87%). The median follow-up was 340 ± 84 days, with 111 (58.1%) patients experiencing a composite endpoint. The average time to the occurrence of the composite endpoint was 110.5 ± 98.7 days. Patients who had MACE were significantly older. There was no statistically significant difference in the incidence of adverse outcomes in patients with ischemic compared to non-ischemic CMP (61.7% vs. 47.7%; *p* = 0.323). During the follow-up period, 30.6% of the patients had cardiovascular deaths, while 69.4% were hospitalized for cardiac decompensation. The patients with MACE had higher percentages of diabetes and renal insufficiency as compared to those without MACE. Additionally, atrial fibrillation was significantly more common in the group of patients with MACE. The laboratory data at discharge showed that the renal function was significantly worse and the level of N-terminal pro-brain natriuretic peptide (NTproBNP) was significantly higher in the group of patients with MACE. Around 80% of the patients received beta-blockers and/or angiotensin-converting enzyme inhibitors/angiotensin-receptor blockers, mineralocorticoid receptor antagonists, and loop diuretics, and this was similar between the groups (Table 1).

In the group of patients with MACE, the echocardiographic findings showed significantly lower LVEF, higher LAVI, and prevalence of moderate/severe MR, lower RV systolic function (FAC, TAPSE, and TAS’), higher TRVmax and PASP, and lower RV-PA coupling parameters (TAPSE/PASP and TAS’/PASP) (Table 2).

The univariate Cox regression analysis of the patients with MACE showed that advanced age, diabetes mellitus, renal insufficiency, higher levels of creatinine and blood urea nitrogen, a lower estimated glomerular filtration rate (eGFR), higher NTproBNP, and atrial fibrillation were associated with the composite endpoint. Additionally, a higher diameter of the left atrium, LAVI, indexed left ventricular mass, indexed left ventricular end-diastolic diameter, a lower LVEF, moderate or severe mitral regurgitation (MR), lower level of systolic right ventricular function (FAC, TAPSE, and TAS’), TAPSE/PASP, and TAS’/PASP, as well as a higher level of tricuspid regurgitation maximum velocity (TRVmax) and a higher estimated pulmonary artery systolic pressure (PASP), were associated with the composite endpoint (Table 3).

The multivariate Cox regression analysis of the parameters that were found to be associated with the composite endpoint identified several independent predictors of adverse outcomes. They were advanced age, atrial fibrillation, LAVI, LVEF, TAPSE/PASP, and TAS’/PASP. TAS’/PASP emerged as the strongest independent predictor of prognosis, with an HR of 0.67 (0.531–0.840), *p* < 0.001 (Table 4).

**Figure 1:**
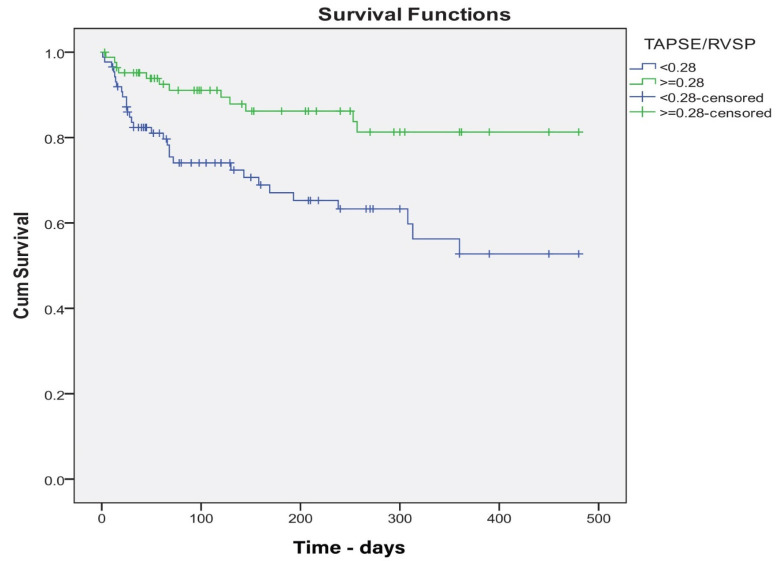
Kaplan–Meier survival curves for prediction of the composite endpoint of the TAPSE/PASP threshold of 0.28.

**Figure 2:**
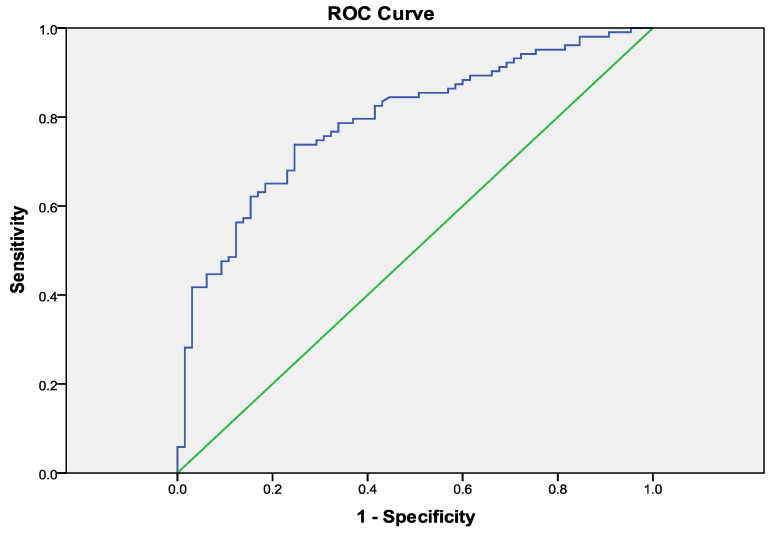
Receiver operating characteristic (ROC) curve analysis for predicting the endpoint with tricuspid annular plane systolic velocity (TAS)/pulmonary arterial systolic pressure (PASP) ratio.

**Figure 3:**
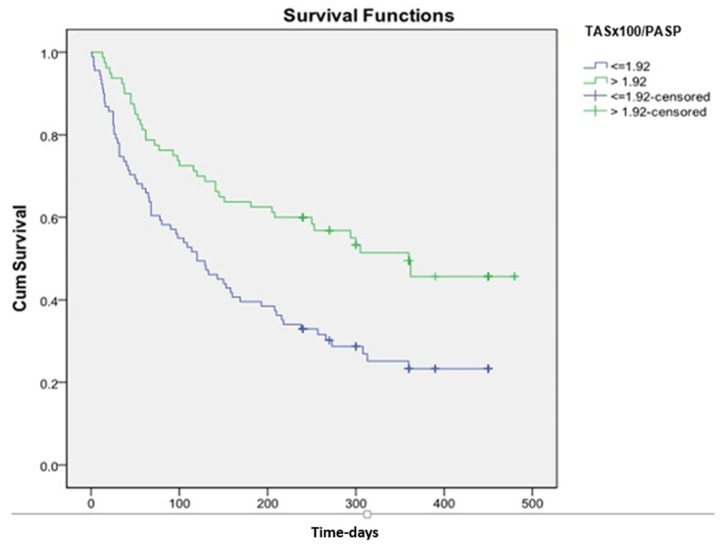
Kaplan–Meier survival curves for prediction of the composite endpoint of the TASx100/PASP threshold of 1.92.

Reconstructing the ROC curve 0.8 (0.723–0.859), *p* < 0.001, we obtained a threshold value of TAS’/PASP ≤ 0.19 (cm/s/mm Hg) (sensitivity 74.0, specificity 75.2). The cut-off for TAPSE/PASP identified by ROC analysis was ≤0.28 mm/mmHg (sensitivity 51.0, specificity 63.4). Patients with TAS’/PASP ≤ 0.19 and TAPSE/PASP ≤ 0.28 had a significantly higher number of MACE (log-rank *p* < 0.001) (Figure 1, Figure 2 and Figure 3).

## 4. Discussion

The main findings of this study were that (I) combined parameters TAS’/PASP and TAPSE/PASP were independent predictors of one-year adverse outcomes, better than each RV echo parameter individually; (II) variable TAS’/PASP proved to be the most powerful, independent predictor of one-year outcome; and (III) to our knowledge, this parameter has not been previously assessed as a predictor of adverse outcome in the HFrEF patients.

This study showed the predictive value of non-invasive echocardiographic parameters of RV-PA coupling for adverse outcomes in patients with heart failure. The diagnostic and prognostic value of the RV-PA coupling in various cardiopulmonary diseases was confirmed in different research [21,23,27,28,29]. Numerous studies have established a correlation between RV dysfunction and adverse outcomes in patients with heart failure. The echocardiographic parameters proposed to measure RV dysfunction have some limitations. For instance, FAC is a challenging index to document and has poor interobserver reproducibility. In contrast, the TAS’ is a simple and reproducible parameter [4,5] and has good predictive value for cardiovascular death and heart failure rehospitalization after discharge in patients with HFrEF and PH [30]. Awad, et al. investigated RV dysfunction in patients diagnosed with ST-elevation myocardial infarction (STEMI) and non-ST-elevation myocardial infarction (NSTEMI) and reported that parameters of RV dysfunction, including the FAC (<37.5%), TAPSE (<15.8 mm), and the TAS’ (<9.67 cm/s), are independent predictors of MACE within the first 30 days after the acute myocardial infarction [31]. Saxena et al. demonstrated that TAS’ correlates well with TAPSE and FAC, parameters usually used to evaluate RV systolic function [32]. The normalization of RV parameters for PASP increased their predictive values. Combined parameters of RV systolic function and loading conditions such as FAC/PASP and TAPSE/PASP are shown to be prognostic factors in patients with HF, PH, and PTE [14,24,25,28]. Li et al. reported that although the gold standard for assessment of RV-PA coupling is invasive RHC, non-invasive echocardiographic surrogates offer new opportunities for assessing RV-PA coupling. The TAPSE/PASP ratio is an easily available and valid parameter in PH used to diagnose a wide range of diseases such as PAH, heart failure, valvular heart disease, pulmonary embolism, etc. [1]. In a large cohort study, patients with mild PH with PASP of 33 to 39 mmHg had reduced RV-PA coupling with a median TAPSE/PASP ratio of 0.55 mm/mmHg, compared with those with PASP < 33 mmHg [27]. The 2022 European Society of Cardiology/European Respiratory Society (ESC/ERS) guideline recommended the TAPSE/PASP cut-off value of 0.55 mm/mmHg as an echocardiographic marker suggestive of PH in the general population. In this recommendation, TAPSE/PASP was shown to have diagnostic value [10]. According to Guazzi et al., the TAPSE/PASP ratio can be used to predict cardiovascular death in patients suffering from both HFrEF and HF with preserved ejection fraction (HFpEF). The researchers identified that patients with a TAPSE/PASP ratio less than 0.36 mm/mmHg had a higher risk of mortality. The cut-off value was similar in both the HFpEF and HFrEF subgroups [21]. In the study conducted by Nakagawa et al., TAPSE/PASP was a prognostically significant factor in predicting adverse outcomes in patients with HFpEF who were acutely decompensated [33]. According to Chen and colleagues, both TAPSE/PASP and TAS’/PASP are closely associated with resting/exercise hemodynamics in patients with HFpEF. They reported that even mild impairment of TAPSE/PASP or TAS’/PASP plays a crucial role in the early detection of PH in patients with HFpEF [34]. TAPSE/PASP and TAS’/PASP may enable the earlier detection of RV-PA uncoupling as an early sign of pulmonary hypertension. This can facilitate the identification of pulmonary hypertension and may have important implications for the early management of HF [35]. Our study found that the parameters of RV-PA coupling, TAS’/PASP, and TAPSE/PASP are independent predictors of adverse outcomes in patients with HFrEF. We observed that TAS’/PASP was the most reliable prognostic parameter with an optimal cut-off value of ≤0.19 and was not previously reported as a predicting parameter of adverse outcomes in HFrEF patients. Further studies are needed to build the best prognostic model in this area. We hope that the results of this study will encourage further investigations into the implications of TAS’/PASP.

### Limitations

The study had some limitations. First, the sample size was relatively small. Second, the RV function was not evaluated by three-dimensional echocardiography. Because the right ventricle has a complex geometry, it is ideal to perform a three-dimensional evaluation. Third, it should be noted that the estimation of systolic pulmonary artery pressure (PSAP) using ultrasound can be unsatisfactory due to inadequate visualization of the inferior vena cava and/or the tricuspid regurgitation velocity continuous wave (CW) Doppler signal. Lastly, it is worth noting that while RHC is the gold standard for comparison and validation, this study was based on non-invasive echocardiographic evaluation.

## 5. Conclusions

One-year rehospitalization and mortality rates were high among patients with HFrEF. Predictors of outcomes identified in this study could aid in risk stratification and timely interventions. This study confirmed previously known predictors of adverse outcomes in patients with HfrEF. They were advanced age, atrial fibrillation, LAVI, and LVEF. However, numerous studies have also demonstrated that implementing non-invasive echo parameters to assess the right ventricle and pulmonary artery coupling can effectively enhance the risk stratification of patients diagnosed with heart failure who have a reduced ejection fraction. Specifically, TAPSE/PASP has been demonstrated to be more effective in predicting adverse outcomes than individual parameters of RV systolic function and loading condition alone. This study also confirmed this result. The novelty of this research is that variable TAS’/PASP emerged as the more potent echocardiography parameter of RV-PA coupling than TAPSE/PASP, serving as an independent predictor of one-year adverse outcomes for patients with HFrEF [36,37].

## Figures and Tables

**Table 1:** Characteristics of the study population, laboratory data, and medications.

Variables	All (*n* 191)	With MACE (*n* 111/191)	Without MACE (*n* 80/191)	*p* Value
Men, *n* (%)	142 (74.2)	83 (58.5)	59 (41.5)	0.872
Age (years)	62.28 ± 12.79	65.0 ± 11.56	57.92 ± 13.21	<0.001
Body mass index (kg/m^2^)	27.80 ± 5.05	28.18 ± 5.52	27.29 ± 4.30	0.529
Comorbidities				
Hypertension, *n* (%)	140 (73.30)	87 (78.4)	53 (66.25)	0.055
Diabetes, *n* (%)	80 (41.88)	54 (67.5)	26 (32.5)	0.026
Hyperlipidemia, *n* (%)	88 (46.07)	53 (47.75)	35 (43.75)	0.584
Obesity, *n* (%)	53 (27.75)	33 (29.7)	20 (25)	0.471
Myocardial infarction, *n* (%)	40 (20.94)	25 (22.5)	15 (18.8)	0.527
Stroke, *n* (%)	27 (14.14)	16 (14.4)	11 (13.8)	0.896
Prior revascularization, *n* (%)	48 (25.13)	28 (25.2)	20 (25.0)	0.200
Atrial fibrillation, *n* (%)	71 (37.17)	53 (47.7)	17 (21.2)	0.002
Renal insufficiency, *n* (%)	92 (48.17)	61 (55.0)	31 (38.8)	0.027
Liver insufficiency, *n* (%)	8 (4.19)	5 (4.5)	3 (3.75)	0.085
Laboratory data at discharge				
Creatinine (μmol/L)	118.14 ± 54.32	125.84 ± 62.14	107.46 ± 39.00	0.021
eGFR (mL/min/1.73 m^2^)	62.91 ± 24.40	59.26 ± 23.10	67.98 ± 25.39	0.014
BUN (mmol/L)	9.90 ± 5.68	11.27 ± 6.61	7.99 ± 3.23	<0.001
Hemoglobin (g/L)	136.77 ± 20.08	135.92 ± 20.15	137.963 ± 20.04	0.489
Albumin (mg/dL)	3.66 ± 0.54	3.62 ± 0.56	3.69 ± 0.52	0.11
CRP (mg/dL)	0.76 ± 1.50	0.85 ± 1.87	0.69 ± 1.12	0.26
NTproBNP	8421.02 ± 6144.90	9653.39 ± 6297.02	6006.59 ± 5333.69	0.007
Medications at discharge				
ACEi/ARBs, *n* (%)	164 (86)	94 (84)	70 (87)	0.4
β-blockers, *n* (%)	160 (84)	93 (83)	67 (84)	0.82
Mineral corticoid-receptor blockers, *n* (%)	137 (72)	81 (73)	56 (70)	0.51
Loop diuretics, *n* (%)	156 (82)	89 (80)	67 (83)	0.33

ACEi, angiotensin-converting enzyme inhibitor; ARB, angiotensin II receptor blocker; eGFR, estimated glomerular filtration rate; BUN, blood urea nitrogen; eGFR, C-reactive protein; NTproBNP, N-terminal pro-brain natriuretic peptide; MACE: major adverse cardiovascular events.

**Table 2:** Echocardiography data.

Variables	All (*n* 191)	With MACE (*n* 111/191)	Without MACE (*n* 80/191)	*p* Value
LVDd-index, mm/m^2^	32.94 ± 5.11	32.95 ± 5.23	32.93 ± 4.96	0.975
LAVI, mL/m^2^	55.90 ± 21.63	58.74 ± 23.94	51.95 ± 17.28	0.024
LVEF, %	25.53 ± 6.87	24.41 ± 6.71	27.08 ± 6.85	0.008
Moderate/severe MR	56 (30%)	28 (25%)	28 (34%)	0.019
FAC (%)	37.50 ± 10.22	36.10 ± 10.55	39.44 ± 9.47	0.026
TAPSE (mm)	15.23 ± 4.32	14.56 ± 4.16	16.16 ± 4.38	0.017
TAS’ (cm/s)	10.4 ± 0.91	9.2 ± 0.27	12.0 ± 1.37	0.039
TRVmax (m/s)	3.00 ± 0.57	3.10 ± 0.52	2.84 ± 0.60	0.003
PASP (mmHg)	50.93 ± 13.31	53.61 ± 12.65	46.67 ± 13.32	<0.001
TAPSE/PASP (mm/mmHg)	0.33 ± 0.16	0.29 ± 0.13	0.38 ± 0.19	<0.001
TAS’/PASP (cm/s/mmHg)	0.21 ± 0.106	0.18 ± 0.09	0.25 ± 0.12	<0.001

LAVI: indexed left atrial systolic volume; LVDd-index: indexed left ventricular end-diastolic diameter; LVEF: left ventricular ejection fraction; MR: mitral regurgitation; FAC: fractional area change; TAPSE: tricuspid annular plane systolic excursion; TAS: tricuspid annular peak systolic velocity; TRVmax: tricuspid regurgitation velocity; PASP: pulmonary arterial systolic pressure.

**Table 3:** The univariate Cox regression analysis of the clinical, laboratory, and echocardiographic parameters.

Parameter	HR	95%CI	*p*
Age (years)	1.03	0.01–1.04	<0.001
Diabetes	1.59	1.09–2.032	0.014
Renal insufficiency	1.60	1.10–2.32	0.013
Atrial fibrillation	2.15	1.46–3.17	<0.001
BUN	1.06	1.03–1.10	<0.001
eGFR	0.99	0.98–0.99	0.007
NTproBNP	1.04	1.01–1.06	0.042
LA	1.04	1.01–1.07	0.004
LAVI	1.01	1.00–1.02	0.003
LVMI	1.005	1.001–1.01	0.028
LVDd-index	1.004	1.001–1.007	0.021
LVEF	0.96	0.94–0.99	0.009
Moderate/severe MR	1.66	1.088–2.521	0.020
FAC	0.97	0.56–0.92	0.005
TAPSE	0.94	0.89–0.98	0.004
TAS’	0.32	0.16–0.63	<0.001
PASP	1.03	1.01–1.04	<0.001
TAPSE/PASP	0.09	0.02–0.36	<0.001
TAS’/PASP	0.32	0.16–0.63	<0.001

BUN, blood urea nitrogen; eGFR, estimated glomerular filtration rate; NTproBNP, an N-terminal pro-brain natriuretic peptide; LA: left atrium; LAVI: indexed left atrial systolic volume; LVMI: indexed left ventricular mass; LVDd-index: indexed left ventricular end-diastolic diameter; LVEF: left ventricular ejection fraction; MR: mitral regurgitation; FAC: fractional area change; TAPSE: tricuspid annular plane systolic excursion; TAS’: tricuspid annular peak systolic velocity; PASP: pulmonary arterial systolic pressure; CI: confidence interval; HR: hazard ratio.

**Table 4:** The multivariate Cox regression analysis.

Covariates	HR	95%CI	*p*
Age	1.028	1.007–1.049	0.01
Atrial fibrillation	1.937	1.013–3.706	0.046
LAVI	1.01	1.004–1.02	0.003
LVEF	0.96	0.94–0.99	0.009
TAPSE/PASP	0.17	0.03–0.93	0.041
TAS’/PASP	0.67	0.53–0.84	<0.001

LAVI: indexed left atrial systolic volume; LVEF: left ventricular ejection fraction; TAPSE: tricuspid annular plane systolic excursion; TAS’: tricuspid annular peak systolic velocity; PASP: pulmonary arterial systolic pressure; CI: confidence interval; HR: hazard ratio.

## Data Availability

The raw data supporting the conclusions of this article will be made available by the authors on request.

## References

[1] Li Q., Zhang M. (2024). Echocardiography assessment of right ventricular-pulmonary artery coupling: Validation of surrogates and clinical utilities. Int. J. Cardiol..

[2] Sanz J., Sánchez-Quintana D., Bossone E., Bogaard H.J., Naeije R. (2019). Anatomy, Function, and Dysfunction of the Right Ventricle: JACC State-of-the-Art Review. J. Am. Coll. Cardiol..

[3] Gorter T.M., van Veldhuisen D.J., Bauersachs J., Borlaug B.A., Celutkiene J., Coats A.J.S., Crespo-Leiro M.G., Guazzi M., Harjola V.P., Heymans S. (2018). Right heart dysfunction and failure in heart failure with preserved ejection fraction: Mechanisms and management. Position statement on behalf of the Heart Failure Association of the European Society of Cardiology. Eur. J. Heart Fail..

[4] Haddad F., Hunt S.A., Rosenthal D.N., Murphy D.J. (2008). Right ventricular function in cardiovascular disease, part I: Anatomy, physiology, aging, and functional assessment of the right ventricle. Circulation.

[5] Haddad F., Doyle R., Murphy D.J., Hunt S.A. (2008). Right ventricular function in cardiovascular disease, part II: Pathophysiology, clinical importance, and management of right ventricular failure. Circulation.

[6] Sanders J.L., Koestenberger M., Rosenkranz S., Maron B.A. (2020). Right ventricular dysfunction and long-term risk of death. Cardiovasc. Diagn. Ther..

[7] Towheed A., Sabbagh E., Gupta R., Assiri S., Chowdhury M.A., Moukarbel G.V., Khuder S.A., Schwann T.A., Bonnell M.R., Cooper C.J. (2021). Right Ventricular Dysfunction and Short-Term Outcomes Following Left-Sided Valvular Surgery: An Echocardiographic Study. J. Am. Heart Assoc..

[8] Benes J., Kotrc M., Wohlfahrt P., Kroupova K., Tupy M., Kautzner J., Melenovsky V. (2023). Right ventricular global dysfunction score: A new concept of right ventricular function assessment in patients with heart failure with reduced ejection fraction (HFrEF). Front. Cardiovasc. Med..

[9] Verhoeff K., Mitchell J.R. (2017). Cardiopulmonary physiology: Why the heart and lungs are inextricably linked. Adv. Physiol. Educ..

[10] Humbert M., Kovacs G., Hoeper M.M., Badagliacca R., Berger R.M.F., Brida M., Carlsen J., Coats A.J.S., Escribano-Subias P., Ferrari P. (2022). 2022 ESC/ERS Guidelines for the diagnosis and treatment of pulmonary hypertension. Eur. Heart J..

[11] Harrison A., Hatton N., Ryan J.J. (2015). The right ventricle under pressure: Evaluating the adaptive and maladaptive changes in the right ventricle in pulmonary arterial hypertension using echocardiography (2013 Grover Conference series). Pulm. Circ..

[12] Vonk Noordegraaf A., Westerhof B.E., Westerhof N. (2017). The Relationship Between the Right Ventricle and its Load in Pulmonary Hypertension. J. Am. Coll. Cardiol..

[13] Tello K., Wan J., Dalmer A., Vanderpool R., Ghofrani H.A., Naeije R., Roller F., Mohajerani E., Seeger W., Herberg U. (2019). Validation of the Tricuspid Annular Plane Systolic Excursion/Systolic Pulmonary Artery Pressure Ratio for the Assessment of Right Ventricular-Arterial Coupling in Severe Pulmonary Hypertension. Circ. Cardiovasc. Imaging.

[14] Guazzi M., Dixon D., Labate V., Beussink-Nelson L., Bandera F., Cuttica M.J., Shah S.J. (2017). RV Contractile Function and its Coupling to Pulmonary Circulation in Heart Failure With Preserved Ejection Fraction: Stratification of Clinical Phenotypes and Outcomes. JACC Cardiovasc. Imaging.

[15] Bashline M.J., Simon M.A. (2019). Use of Tricuspid Annular Plane Systolic Excursion/Pulmonary Artery Systolic Pressure As a Non-Invasive Method to Assess Right Ventricular-PA Coupling in Patients With Pulmonary Hypertension. Circ. Cardiovasc. Imaging.

[16] Parasuraman S., Walker S., Loudon B.L., Gollop N.D., Wilson A.M., Lowery C., Frenneaux M.P. (2016). Assessment of pulmonary artery pressure by echocardiography-A comprehensive review. Int. J. Cardiol. Heart Vasc..

[17] Todaro M.C., Carerj S., Zito C., Trifirò M.P., Consolo G., Khandheria B. (2020). Echocardiographic evaluation of right ventricular-arterial coupling in pulmonary hypertension. Am. J. Cardiovasc. Dis..

[18] Ishiwata J., Daimon M., Nakanishi K., Sugimoto T., Kawata T., Shinozaki T., Nakao T., Hirokawa M., Sawada N., Yoshida Y. (2021). Combined evaluation of right ventricular function using echocardiography in non-ischaemic dilated cardiomyopathy. ESC Heart Fail..

[19] Lang R.M., Badano L.P., Mor-Avi V., Afilalo J., Armstrong A., Ernande L., Flachskampf F.A., Foster E., Goldstein S.A., Kuznetsova T. (2015). Recommendations for cardiac chamber quantification by echocardiography in adults: An update from the American Society of Echocardiography and the European Association of Cardiovascular Imaging. J. Am. Soc. Echocardiogr..

[20] Rudski L.G., Lai W.W., Afilalo J., Hua L., Handschumacher M.D., Chandrasekaran K., Solomon S.D., Louie E.K., Schiller N.B. (2010). Guidelines for the echocardiographic assessment of the right heart in adults: A report from the American Society of Echocardiography endorsed by the European Association of Echocardiography, a registered branch of the European Society of Cardiology, and the Canadian Society of Echocardiography. J. Am. Soc. Echocardiogr..

[21] Guazzi M., Bandera F., Pelissero G., Castelvecchio S., Menicanti L., Ghio S., Temporelli P.L., Arena R. (2013). Tricuspid annular plane systolic excursion and pulmonary arterial systolic pressure relationship in heart failure: An index of right ventricular contractile function and prognosis. Am. J. Physiol. Heart Circ. Physiol..

[22] Fauvel C., Raitiere O., Boucly A., De Groote P., Renard S., Bertona J., Lamblin N., Artaud-Macari E., Viacroze C., Schleifer D. (2022). Interest of TAPSE/sPAP ratio for noninvasive pulmonary arterial hypertension risk assessment. J. Heart Lung Transplant..

[23] Gorter T.M., van Veldhuisen D.J., Voors A.A., Hummel Y.M., Lam C.S., Berger R.M., van Melle J.P., Hoendermis E.S. (2018). Right ventricular-vascular coupling in heart failure with preserved ejection fraction and pre- vs. post-capillary pulmonary hypertension. Eur. Heart J. Cardiovasc. Imaging.

[24] Guazzi M., Villani S., Generati G., Ferraro O.E., Pellegrino M., Alfonzetti E., Labate V., Gaeta M., Sugimoto T., Bandera F. (2016). Right Ventricular Contractile Reserve and Pulmonary Circulation Uncoupling During Exercise Challenge in Heart Failure: Pathophysiology and Clinical Phenotypes. JACC Heart Fail..

[25] Tello K., Axmann J., Ghofrani H.A., Naeije R., Narcin N., Rieth A., Seeger W., Gall H., Richter M.J. (2018). Relevance of the TAPSE/PASP ratio in pulmonary arterial hypertension. Int. J. Cardiol..

[26] Felker G.M., Shaw L.K., O’Connor C.M. (2002). A standardized definition of ischemic cardiomyopathy for use in clinical research. J. Am. Coll. Cardiol..

[27] Huston J.H., Maron B.A., French J., Huang S., Thayer T., Farber-Eger E.H., Wells Q.S., Choudhary G., Hemnes A.R., Brittain E.L. (2019). Association of Mild Echocardiographic Pulmonary Hypertension with Mortality and Right Ventricular Function. JAMA Cardiol..

[28] Siddiqui I., Rajagopal S., Brucker A., Chiswell K., Christopher B., Alenezi F., Mandawat A., Rivera D., Arges K., Tapson V. (2018). Clinical and Echocardiographic Predictors of Outcomes in Patients with Pulmonary Hypertension. Am. J. Cardiol..

[29] Meluzín J., Špinarová L., Bakala J., Toman J., Krejčí J., Hude P., Kára T., Souček M. (2001). Pulsed Doppler tissue imaging of the velocity of tricuspid annular systolic motion; a new, rapid, and non-invasive method of evaluating right ventricular systolic function. Eur. Heart J..

[30] Saito C., Jujo K., Kametani M., Arai K., Fukushima N., Minami Y., Abe T., Takagi A., Ashihara K., Hagiwara N. (2022). Prognostic impact of right ventricular function affected by pulmonary hypertension in hospitalized heart failure patients. J. Cardiol..

[31] Awad E.M.L., Mahmoud A.H., Maghrby K.S., Taha N.M., Ibrahim A.M. (2020). Short-term prognostic value of TAPSE, RVFAC and Tricuspid S’ wave peak systolic velocity after first acute myocardial infarction. BMC Res. Notes..

[32] Saxena N., Rajagopalan N., Edelman K., López-Candales A. (2006). Tricuspid annular systolic velocity: A useful measurement in determining right ventricular systolic function regardless of pulmonary artery pressures. Echocardiography.

[33] Nakagawa A., Yasumura Y., Yoshida C., Okumura T., Tateishi J., Yoshida J., Abe H., Tamaki S., Yano M., Hayashi T. (2020). Prognostic Importance of Right Ventricular-Vascular Uncoupling in Acute Decompensated Heart Failure With Preserved Ejection Fraction. Circ. Cardiovasc. Imaging.

[34] Chen Z., Chung Y., Cheng J., Huang C., Chen S., Lin L., Lai H., Wu C. (2024). Right Ventricular-Vascular Uncoupling Predicts Pulmonary Hypertension in Clinically Diagnosed Heart Failure with Preserved Ejection Fraction. J. Am. Heart Assoc..

[35] Badagliacca R., Ghio S., Manzi G., Vizza C.D. (2024). Right Ventricular/Pulmonary Artery Coupling in Patients With Heart Failure With Preserved Ejection Fraction: A Clue for Pulmonary Hypertension?. J. Am. Heart Assoc..

[36] Tay W.T., Teng T.K., Simon O., Ouwerkerk W., Tromp J., Doughty R.N., Richards A.M., Hung C., Qin Y., Aung T. (2021). ASIAN-HF Investigators. Readmissions, Death and Its Associated Predictors in Heart Failure With Preserved Versus Reduced Ejection Fraction. J. Am. Heart Assoc..

[37] Parikh R.V., Go A.S., Bhatt A.S., Tan T.C., Allen A.R., Feng K.Y., Hamilton S.A., Tai A.S., Fitzpatrick J.K., Lee K.K. (2023). Developing Clinical Risk Prediction Models for Worsening Heart Failure Events and Death by Left Ventricular Ejection Fraction. J. Am. Heart Assoc..

